# Body representation underlies response of proprioceptive acuity to repetitive peripheral magnetic stimulation

**DOI:** 10.3389/fnhum.2022.924123

**Published:** 2022-08-09

**Authors:** Yunxiang Xia, Kento Tanaka, Man Yang, Shinichi Izumi

**Affiliations:** ^1^Department of Physical Medicine and Rehabilitation, Graduate School of Medicine, Tohoku University, Sendai, Japan; ^2^Graduate School of Dalian Medical University, Dalian, China

**Keywords:** body representation, proprioceptive acuity, repetitive peripheral magnetic stimulation, body image, body schema

## Abstract

Proprioceptive acuity is of great significance in basic research exploring a possible neural mechanism of fine motor control and in neurorehabilitation practice promoting motor function recovery of limb-disabled people. Moreover, body representation relies on the integration of multiple somatic sensations, including proprioception that is mainly generated in muscles and tendons of human joints. This study aimed to examine two hypotheses: First, different extension positions of wrist joint have different proprioceptive acuities, which might indicate different body representations of wrist joint in the brain. Second, repetitive peripheral magnetic stimulation (rPMS) applied peripherally to the forearm radial nerve and extensors could change proprioceptive acuity at the wrist joint. Thirty-five healthy participants were recruited then randomly divided into the real stimulation group (*n* = 15) and the sham stimulation group (*n* = 20). The participants’ non-dominant side wrist joint position sense was tested at six extension positions within the physiological joint motion range (i.e., 10°, 20°, 30°, 40°, 50°, 60°) both before stimulation and after stimulation. Results showed that proprioceptive bias (arithmetic difference of target position and replicated position) among six extension positions could be divided into lower-extension position (i.e., 10°, 20°, 30°) and higher-extension position (i.e., 40°, 50°, 60°). One session rPMS could influence proprioceptive bias in lower-extension position but not in higher-extension position. However, proprioceptive precision (standard deviation within lower-extension position and higher-extension position) was not influenced. To conclude, proprioceptive bias may vary between different wrist extension positions due to different hand postures being related to changes in body representation, and different functions relating to proprioceptive bias and proprioceptive precision may underlie two aspects of body representation.

## Introduction

Proprioception coming from proprioceptors located in joints, muscles, tendons, and skin is encoded biomechanically then transmitted to the central nervous system, which carries out multi-sensory association, including an efference copy, allowing individuals to be aware of their body posture, position in space, movements, and updating body representation (Morasso et al., 2015; Proske, 2019; Albanese et al., 2021). Proprioception can be divided into two modalities: joint position sense and kinesthesia; the former refers to the ability of the subject to perceive a presented joint position and replicate it without the aid of vision, while the latter refers to the ability to perceive movements of the subject’s own body. Joint position sense is the most studied among the two modalities, with previous research demonstrating that proprioceptive acuity of joint position sense is impaired after physical injury, osteoarthritis, as well as in healthy elderly and chronic patients with post-stroke (Felson et al., 2009; Goble et al., 2009; Langhorne et al., 2009; Van de Winckel et al., 2017; Mugnosso et al., 2019; Fisher et al., 2020). To explore the effectiveness of rehabilitation protocols so as to benefit motor recovery of these populations, investigation of joint position sense is necessary to clarify the plasticity of proprioceptive acuity and its possible relationship with body representation secondary to a motor outcome (Masia et al., 2009; De Santis et al., 2015; Albanese et al., 2021). Even though ways like identification of imposed movement direction and measurement of a movement detection threshold are available for assessing proprioceptive acuity, more and more research is prone to utilize joint position matching as a more useful tool with acceptable validity and reliability (Zia et al., 2002; Felson et al., 2009; Elangovan et al., 2014). Joint position matching also could be seen in rehabilitation practice nowadays for the growing understanding of the role of inputting sensory information, especially proprioception, which plays in promoting neural plasticity through a use-dependent mechanism (Nudo, 2007; Goble, 2010; Oouchida et al., 2016).

There are usually two conditions of joint position matching: one is the ipsilateral joint position matching in which the participant replicates target joint position, given by an experimenter; the other is the contralateral joint position matching whereby the participant matches target joint position with the counterpart joint (Van de Winckel et al., 2017). We adopted the ipsilateral wrist joint position matching design for this study because it involves generating internal body representation and possible higher accuracy (Elangovan et al., 2014; Holst-Wolf et al., 2016). We argue that the perceived impression of target position of one’s joint does not fully equal to the commonly defined term “memory,” which refers to stored information in the past (Goble, 2010; Elangovan et al., 2014). However, the ipsilateral joint position matching might be related to working memory, which is a cognitive system of limited capacity that holds information temporarily (Elangovan et al., 2014). The participant in this kind of experiment is asked to focus on perceiving the spatial position of an occluded joint in a very short period, usually a few seconds, whose process is closer to the connotation of body representation that is a neural representation of the body parts relative to each other and one’s knowledge and belief of his or her own body (Corbett and Shah, 1996; de Vignemont, 2010; Longo and Haggard, 2010; Medina and Coslett, 2010; Assaiante et al., 2014; Vita et al., 2016; Llorens et al., 2017; Pitron and de Vignemont, 2017; Pitron et al., 2018; Gadsby, 2019; Leemhuis et al., 2019; Di Vita et al., 2020; D’Amour and Harris, 2020; Raimo et al., 2021). Regarding the codification mechanism of proprioceptive acuity, some of the previous research has suggested it is highly correlated with joint position (Marini et al., 2017b,^2018^), while some evidence suggested it is amplitude based rather than position based (Marini et al., 2016). Whichever it is amplitude based, or position based, from the perspective of body representation, they both are body posture concerned. Specifically, the former is dynamic body posture, while the latter is static body posture. Body representation that relied mainly on proprioception might imply one possible neural mechanism of proprioceptive acuity (Marini et al., 2019). Moreover, latest research elucidated that two similar but rotated hand postures were related to different body representations: forearm pronated, thumb down, index up pinch compared with the same grip performed with the thumb up, in which the former revealed a faster movement onset, a sign of faster neural computation, faster target reaching, and increased corticospinal excitability, which suggested the existence of a baseline postural representation that may serve as *a priori* spatial reference for body–space interaction (Romano et al., 2021). Based on these findings, our first hypothesis is that different extension positions of wrist joint might have different body representations as well, which, in the experimental paradigm of ipsilateral wrist joint position matching, there might be one or more certain positions in the ballistic extension movement that could influence proprioceptive acuity.

Repetitive peripheral magnetic stimulation (rPMS) is widely used in clinical practice nowadays to reduce spasticity and improve the ability of daily living of upper limb and hand dexterity (Struppler et al., 2003b; Chen et al., 2020). rPMS applied to the peripheral nerve induces proprioceptive inflow to the central nervous system (CNS) in two different ways: adequate activation (indirectly due to stimulation) of mechanoreceptors (fiber groups Ia, Ib, II) during the rhythmic contraction and relaxation, as well as vibration of the muscles; inadequate activation (directly due to the stimulation) of sensorimotor nerve fibers with orthodromic and antidromic conduction (Struppler et al., 2004; Behrens et al., 2011; Beaulieu and Schneider, 2013). The direct relationship between rPMS and joint motor control remains less known; however, given the significance of neural mechanisms of motor function recovery, it is relevant to establish the effect of rPMS on response of proprioceptive acuity in healthy adults. Nito et al. (2021) examined the effects of rPMS over wrist extensor muscles on neural plasticity and motor performance in healthy volunteers, in which significant increase in motor-evoked potentials (MEPs) was observed, but the maximal M-wave and Hoffmann-reflex did not change, suggesting the plastic changes at the motor cortex. Since rPMS could input proprioception inflow to CNS, and proprioception contributes to body representation, combining with the former hypothesis, we further hypothesized that proprioceptive acuity of wrist joint position sense might be influenced differently at different extension positions by rPMS. In fact, Struppler et al. conducted a similar experiment that they found rPMS could improve elbow joint position sense of a certain position (Struppler et al., 2003a). We extended their work by replacing the elbow joint to wrist joint and set up six gradient extension positions within a physiological joint range to explore possible body representation diversities.

## Materials and methods

### Experimental design

We performed this cross-sectional study, which was to test whether proprioceptive acuities of wrist joint position sense were different at different extension positions and whether rPMS could influence proprioceptive acuity differently at different extension positions. In order to achieve these goals, we set up two matched groups in healthy young adults: the real stimulation group and the sham stimulation group. First, we examined whether proprioceptive acuities in ipsilateral wrist joint position matching were different at different extension positions, for this replicating method was to measure the same body representation or working memory of the target position (Elangovan et al., 2014). Therefore, taken the neutral position of the wrist joint as the start position, we set up 6 extension positions: 10°, 20°, 30°, 40°, 50°, 60°. Proprioceptive acuity was tested two times at every extension position whose sequence was randomized beforehand, before and after sham or real stimulation. Next, by comparing performance of after stimulation adjusted by performance of pre-stimulation in two groups, it was supposed to figure out whether rPMS can exert any effect on proprioceptive acuity.

### Measurements

When envisioning proprioceptive acuity of joint position sense, it can be interpreted as proprioceptive bias and proprioceptive precision. Proprioceptive bias is calculated by subtracting the angle of replicated position from that of the target position, indicating how close a perceived joint position corresponds to the true physical position; therefore, negative values indicate replicated position overshoots target position. We also used the absolute value of the difference between target joint position and replicated joint position calculated above as an alternative index to explore which one is more appropriate for representing proprioceptive bias. Proprioceptive precision is calculated by using the standard deviation of repeated measurements of proprioceptive bias, reflecting variability of joint position sense.

### Participants

Sample size was determined by the G*power (version 3.1.9.7). Considering the possible statistical methods for analysis (details described below), first, we chose F tests, ANOVA (Fixed effects, omnibus, one-way), *a priori* type of power analysis, effect size of 0.5, an err prob of 0.05, power of 0.8; number of groups, 2; calculation of total sample size was 34. Second, we chose F tests, ANCOVA (Fixed effects, main effects, and interactions), *a priori* type of power analysis, effect size of 0.5, an err prob of 0.05, power of 0.8; number of groups, 2; number of covariates, 1; calculation of total sample size was also 34. Therefore, 35 right-handed participants (age = 25.7 ± 2.9 years, 19 men) were recruited through posters in the campus of Tohoku University and randomly divided them into the real stimulation group and the sham stimulation group, with all participants blinded to this information. Fifteen participants in the real stimulation group (age = 24.5 ± 1.8 years, 9 men), and 20 participants in the sham stimulation group (age = 26.6 ± 3.3 years, 10 men). No statistical differences were found in age and gender between groups. All participants were asked about sequelae from muscular and neurological diseases or injuries. The participants were included when they were asymptomatic, with no history of any type of injury or wrist joint instability, no cognitive disability, sensory loss, or motor impairment. The participants were excluded if they had the following conditions: (1). could not move their wrist joints without pain in a full range of motion or feel any inconvenience during such wrist joint movements; (2). the wrist joint involved expertise, such as athletes of basketball or baseball, and any kind of handicraft; (3). the active and passive extension range wrist joint was smaller than the normal range (0°∼70°).

### Apparatus

**FIGURE 1:**
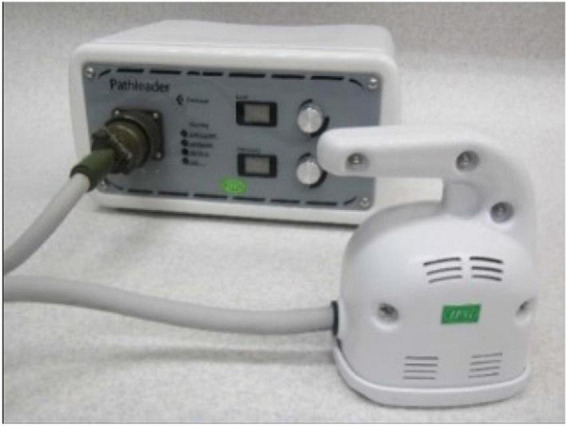
A photo of a repetitive peripheral magnetic stimulation device.

The rPMS device used in this study [Commercial Name: Pathleader, IFG Co., Ltd, Japan Medical Device Number (Type II): 36902000, Japan Medical Device Identification Code: 227AFBZX00021000], consisted of a generator and a round coil that had a cube appearance. The diameter of the coil was 10 cm. A dual-channel electrical goniometer (SG150) made by Biometrics Company was used for collecting real-time angle information, which was converted into analyzable data by LabChart Lightning (ADInstruments Company). See Figure 1.

### Experimental procedures

**FIGURE 2:**
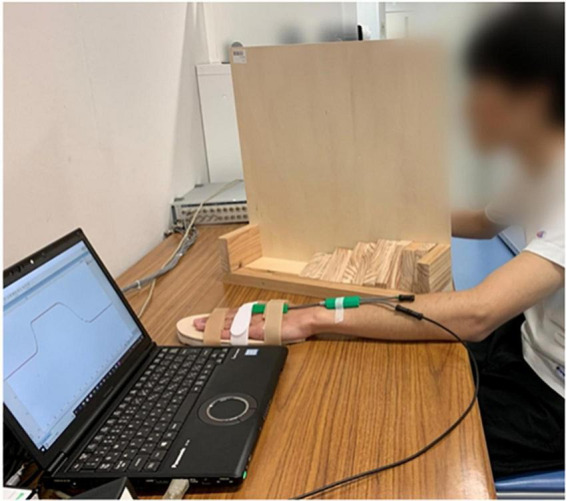
Experimental settings, consisted of an experimental table, a laptop installed with LabChart Lightning and connected to the electrical goniometer, and a vertical partition. The participant was undergoing ipsilateral wrist joint position matching with his non-dominant side wrist placed in neutral position.

The participants were asked to sit in front of a table comfortably, their non-dominant side upper limbs placed flat on a table. We chose the non-dominant side upper limb for it might have greater potential to be improved by rPMS compared to dominant side (Xia et al., 2022). The forearm should be pronated, while wrist joint placed in neutral position. A hand-shaped wooden board was attached to participants’ non-dominant side palms to fix the fingers and make the palms flat for the sake of reducing measurement error. Electrical goniometer was fixed firmly onto the wrist joint using adhesive tape, with the spring sensor vertical to the baseline of the wrist joint. The participants’ vision was blocked by a vertical partition. See Figure 2. The experimenter moved the participants’ wrist to a target extension position slowly and steadily at the speed of 10°/s according to the converted angle curve shown in the computer screen. When arrived at the target position, the experimenter reminded the participants to perceive this position and keep in that position for 5 s. After that, the experimenter moved the participants’ wrist joints back to the neutral position and asked the participants to replicate that target position. This process repeated one more time for the same target position after resting for 5 s. There was 1-min rest time between every two target positions. After all the 6 target positions were tested, the participants underwent one session real rPMS or sham rPMS protocols that included 36 stimulation cycles in total (Schneider et al., 2022). Parameters for the rPMS device were 50–80% of the maximum intensity, frequency of 50 Hz, stimulation time of 2 s and stimulation interval of 1 s (Xia et al., 2022). There was 10-min resting time between the completion of stimulation protocols and the second testing procedures of proprioceptive acuity to avoid the possible confounding effect of fatigue and learning effect. In the real stimulation group, the center of the coil was placed on the outer upper side of the forearm where beneath was the starting segment of the motor branch of the radial nerve to activate the extensor carpi radialis, as it has been reported that motor dysfunction was often observed after corticospinal tract lesions, such as following stroke (Nito et al., 2021). It might take a few trials before the real stimulation begins to find out the proper area that could induce obvious extension movements of the wrist joint and fingers. In the sham stimulation group, the experimenter rotated the cube-shaped coil to 90°, while the rest of the protocols kept the same with the real stimulation group.

### Statistical analysis

To elucidate the potential difference of proprioceptive acuities among 6 extension positions of wrist joint, we first adopted one-way ANOVA (analysis of variance) for both proprioceptive bias and absolute value of proprioceptive bias, with multiple comparisons as the *post hoc* test (Bonferroni’s correction). When performing this analysis, we used both the two values for every six extension positions. Next, we divided 6 extension positions into the lower-extension position (i.e., 10°, 20°, 30°) and the higher extension position (40°, 50°, 60°) according to the results of one-way ANOVA. In the lower-extension position, all three positions were measured two times; therefore, taken the lower angle group as one single position, there should be 6 repeated measurements. It was the same in the higher angle group. Proprioceptive bias and proprioceptive precision were calculated and used for elucidating whether rPMS had an effect on proprioceptive acuity. To avoid the pre-stimulation imbalance, we took the value of pre-stimulation as covariate and performed one-way ANCOVA (analysis of covariance) for the lower-extension position and the higher-extension position, respectively. Analysis was conducted by SPSS (IBM company, version 26). Continuous variables were presented as Mean ± standard Error. *P* < 0.05 was considered to indicate statistical significance.

## Results

### Proprioceptive bias varied in different extension positions

**FIGURE 3:**
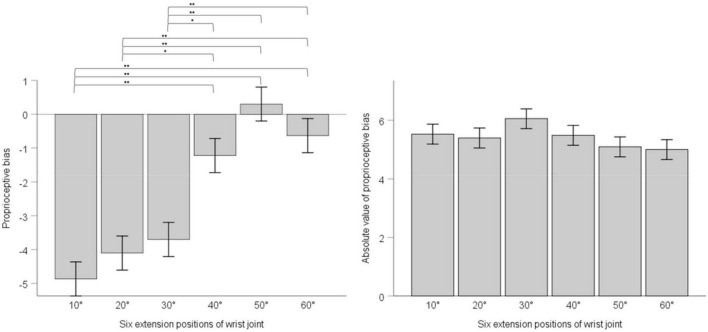
The left panel shows proprioceptive bias in six extension positions of the wrist joint; the right panel shows the absolute value of proprioceptive bias in six extension positions of the wrist joint. Error bars indicate Standard Error. ** indicates *p* < 0.001. * indicates *p* < 0.01.

We adopted one-way ANOVA for analyzing whether there was difference of proprioceptive bias or the absolute value of proprioceptive bias among different extension positions. Results showed that position’s main effect on proprioceptive bias had statistical significance, *F* (5, 30) = 17.63, *p* < 0.001, ηp2 = 0.096. Multiple comparisons with the Bonferroni’s correction are as below. 10° compared with 20°, mean difference = -0.76, 95% confidence interval (−2.87 ∼ 1.34), *p* = 1; 10° compared with 30°, mean difference = −1.17, 95% confidence interval (−3.27 ∼0.94), *p* = 1; 10° compared with 40°, mean difference = −3.64, 95% confidence interval (−5.74 ∼−1.54), *p* < 0.001; 10° compared with 50°, mean difference = −5.17, 95% confidence interval (−7.27 ∼−3.07), *p* < 0.001; 10° compared with 60°, mean difference = −4.23, 95% confidence interval (−6.33 ∼−2.13). 20° compared with 30°, mean difference = −0.40, 95% confidence interval (−2.50 ∼ 1.70), *p* = 1; 20° compared with 40°, mean difference = −2.89, 95% confidence interval (−4.98 ∼−0.78), *p* = 0.001; 20° compared with 50°, mean difference = −4.40, 95% confidence interval (−6.50 ∼−2.30), *p* < 0.001; 20° compared with 60°, mean difference = −3.47, 95% confidence interval (−5.57 ∼−1.36), *p* < 0.001. 30° compared with 40°, mean difference = −2.48, 95% confidence interval (−4.58 ∼−0.38), *p* = 0.008; 30° compared with 50°, mean difference = −4.00, 95% confidence interval (−6.10 ∼−1.90), *p* < 0.001; 30° compared with 60°, mean difference = −3.06, 95% confidence interval (−5.17 ∼−0.96), *p* < 0.001. 40° compared with 50°, mean difference = −1.53, 95% confidence interval (−3.63 ∼0.58), *p* = 0.493; 40° compared with 60°, mean difference = −0.59, 95% confidence interval (−2.69 ∼−1.51), *p* = 1.50° compared with 60°, mean difference = 0.94, 95% confidence interval (−1.16 ∼−3.04), *p* = 1. Position’s main effect on the absolute value of proprioceptive bias was not statistically significant, F (5, 30) = 1.217, *p* = 0.299, ηp2 = 0.007. See Figure 3.

### Effect of repetitive peripheral magnetic stimulation on proprioceptive bias

**FIGURE 4:**
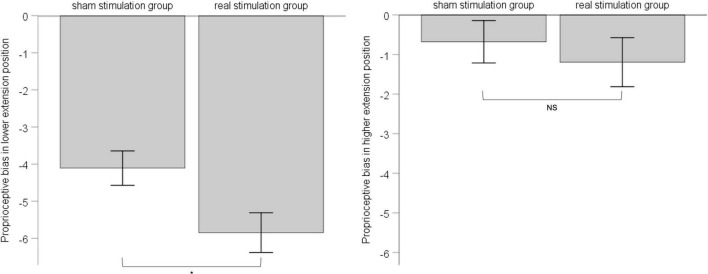
The left panel shows post-stimulation performance of proprioceptive bias in the lower-extension position; the right panel shows post-stimulation performance of proprioceptive bias in the higher-extension position. Error bars indicate Standard Error. * indicates *p* < 0.05. NS indicates no statistical significance.

According to the result of position’s main effect on proprioceptive bias in ANOVA, we divided 6 extension positions into lower-extension position (i.e., 10°, 20°,30°) and higher-extension position (i.e., 40°, 50°, 60°). For either the lower-extension position or the higher-extension position, there were six measurements in total, and we took the corresponding six measurements as proprioceptive bias of the lower-extension position or the higher-extension position. Considering the influence of pre-stimulation difference between the sham stimulation group and the real stimulation group, we chose one-way ANCOVA with pre-stimulation performance of proprioceptive bias as covariate to explore the effect of rPMS. Results showed that, in the lower-extension position, pre-stimulation performance of proprioceptive bias was adjusted as -3.60, had effect on post-stimulation performance of proprioceptive bias, *F* (1, 34) = 28.197, *p* < 0.001, ηp2 = 0.12; the sham stimulation group compared with the real stimulation group, mean difference = −4.11, 95% confidence interval (−5.02 ∼−3.19), had statistical significance, *F* (1, 34) = 6.017, *p* = 0.015, ηp2 = 0.028. In the higher-extension position, pre-stimulation performance of proprioceptive bias was adjusted as −0.14, had effect on post-stimulation performance of proprioceptive bias, *F* (1, 34) = 43.69, *p* < 0.001, ηp2 = 0.174; the sham stimulation group compared with the real stimulation group, mean difference = −0.68, 95% confidence interval (−1.74 ∼−0.38), had no statistical significance, *F* (1, 34) = −0.397, *p* = 0.53, ηp2 = 0.002. See Figure 4.

### Effect of repetitive peripheral magnetic stimulation on proprioceptive precision

**FIGURE 5:**
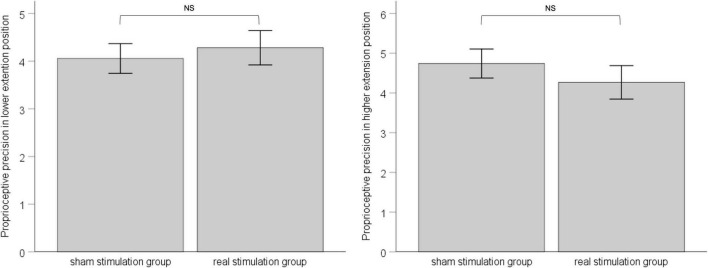
The left panel shows post-stimulation performance of proprioceptive precision in the lower-extension position; the right panel shows post-stimulation performance of proprioceptive precision in the higher-extension position. Error bars indicate Standard Error. NS indicates no statistical significance.

In both the lower-extension position and the higher-extension position, we calculated standard deviation of six measurements for each, as proprioceptive precision. ANCOVA was applied with pre-stimulation performance of proprioceptive precision as covariate to eliminate the influence of individual difference between groups. Results showed that, in the lower-extension position, pre-stimulation performance of proprioceptive precision was adjusted as −4.69, had effect on post-stimulation performance of proprioceptive precision, *F* (1, 34) = 4.133, *p* = 0.05, ηp2 = 0.114; the sham stimulation group compared with the real stimulation group, mean difference = 4.06, 95% confidence interval (3.42 ∼ 4.69), had no statistical significance, F (1, 34) = 0.222, *p* = 0.64, ηp2 = 0.007. In the higher-extension position, pre-stimulation performance of proprioceptive precision was adjusted as 4.57, had no effect on post-stimulation performance of proprioceptive precision, *F* (1, 34) = 3.192, *p* = 0.083, ηp2 = 0.091; the sham stimulation group compared with the real stimulation group, mean difference = 4.74, 95% confidence interval (4.00 ∼ 5.48), had no statistical significance, *F* (1, 34) = 0.715, *p* = 0.40, ηp2 = 0.022. See Figure 5.

## Discussion

This study tested two hypotheses. First, different extension positions of the wrist joint have different proprioceptive acuities, which might indicate different body representations of the wrist joint in the brain. Second, rPMS could influence proprioceptive bias in the lower-extension position but not the higher-extension position; moreover, rPMS could not influence proprioceptive precision; the observed results might be interpreted by the current understanding of different aspects of body representation. Further discussion is as below.

From the analysis result of different extension positions on proprioceptive bias, we could easily divide six extension positions into the lower-extension position (i.e., 10°, 20°, 30°) and the higher-extension position (i.e., 40°, 50°, 60°). Proprioceptive bias in the lower-extension position tended to overshoot target position, while in the higher-extension position was relatively accurate. In fact, Francesca et al. found that performance of wrist proprioceptive bias was highly correlated with the starting position and targets tended to be overshot when the active matching movements were longer (Marini et al., 2017b). Combined with this study, the starting position might be a more important factor regarding the overshoot characteristic of active matching. In the lower-extension-position where the wrist joint rested at the neutral position, has relatively more space in the ballistic extension movement compared to the higher-extension position; thus, active matching tends to move forward than the target position. We propose that the position representation of the lower-extension position in the brain might be weaker than that of the higher-extension position; therefore, proprioceptive bias in the higher-extension position was more accurate than in the lower-extension position. It is consistent with the research mentioned in the introduction that, in two pinch postures, a baseline postural representation may serve as *a priori* spatial reference for interacting with outer environment. We also supplement one possible reason for this phenomenon, that the amount and the variety of proprioceptive generators initiated in different body postures were different, and cortex areas corresponding to these proprioceptive generators might also be different; therefore, the higher-extension position whose maintenance involved more muscles and ligaments than the near neutral position of the lower-extension position has a stronger body representation.

The absolute value of proprioceptive bias in six extension positions was not statistically different; therefore, we did not use this index for further analysis. The absolute value ignored the direction of error between the target position and replicated position, namely, overshooting and undershooting the same extent of the target position make no difference. However, in the discussion above, we deduced that the overshooting characteristic of the lower-extension position might imply some neural mechanism of body representation of the wrist joint, which is worth further investigations in the future.

Next, we found that rPMS influenced proprioceptive bias in the lower-extension position but not in the higher-extension position. Specifically, real rPMS made replicated position overshot more than sham rPMS in the lower-extension position. As stated before, rPMS applied to peripheral nerve inputted proprioception and proprioception that contribute to body representation. One-session real rPMS could influence the weaker body representation of the lower-extension position but failed to show obvious effect on the higher-extension position with statistical significance. However, we did observe a similar overshooting phenomenon in the higher-extension position in this study; multiple sessions of rPMS study are needed to verify this point.

Except for the influence on proprioceptive bias, rPMS did not show effect on proprioceptive precision in neither the lower-extension position nor the higher-extension position, which was in accordance with previous research that demonstrated a similar effect of aging, that, for ipsilateral joint position matching of the wrist joint, old adults compared with young adults – their proprioceptive bias increased but proprioceptive precision was not changed (Van de Winckel et al., 2017). From the perspective of the physiological function decline of the human body, including proprioception deterioration of joints caused by aging, proprioceptive bias increase could be reasonably explained. What is more, aging generally means integrating more experience into the subject by utilizing his or her own body; however, this does not necessarily return beneficial or harmful feedback to the subject, except for some rare cases like sports elites or other professionals. Therefore, we propose that proprioceptive bias and proprioceptive precision reflect different aspects of body representation. Proprioceptive bias is closely related to the aspect of the body schema, which refers to relative position in space of body parts relied on real-time proprioception input (de Vignemont, 2010; Medina and Coslett, 2010; Pitron et al., 2018); while proprioceptive precision is related to the other aspect of body representation, body image, which is the accumulative experience of the subject’s own body (de Vignemont, 2010; Pitron and de Vignemont, 2017; Gadsby, 2019). This interpretation could be verified by another research, exploring elbow joint position sense between early childhood and adulthood. What they found was an age-related improvement in proprioceptive precision but not a development or change in proprioceptive bias (Holst-Wolf et al., 2016). Proprioception between children and young adults does not have significant difference, but it probably declines due to peripheral and central changes when running into old age (Ribeiro and Oliveira, 2007; Goble et al., 2009). However, experience of using our body increases from childhood to adulthood and then remains steady, which might underlie the steady performance of proprioceptive precision. On the contrary, children and young adults with probable developmental coordination disorder, which indicated impaired or inadequate experience compared with healthy peers, exhibited significantly increased proprioceptive precision (Tseng et al., 2018, 2022). Besides, Mugnosso et al. revealed that muscle fatigue, which inputted abundance of proprioception, could decrease proprioceptive bias but not proprioceptive precision (Mugnosso et al., 2019). However, external forces that might not be able to input enough proprioception as the repeated active movements of what fatigue protocol achieved failed to affect proprioceptive bias (Kuling et al., 2013; Marini et al., 2017a). Combined with this study, proprioception input from distal upper extremity induced directly by rPMS and indirectly by extension movements of the wrist joint could only improve proprioceptive bias but did not influence the experience of joint position matching; therefore, proprioceptive precision remained unchanged.

Overall, proprioceptive bias in the lower-extension position differed from that in the higher-extension position and could be influenced by rPMS. We interpreted this result as it might be body representation that underlied this observed effect. In a relevant study of our recent work, we used the same rPMS protocol and found directly that implicit body representation of the hand could be enlarged within the boundary of the participant’s real hand (Xia et al., 2022). Similar to rPMS, another intervention of transcutaneous electrical nerve stimulation (TENS), which implanted electrodes that generate proprioception in missing limbs of amputees, has also been found related to body representation; moreover, TENS paresthesia projected into an artificial limb can enhance the sense of perceptual embodiment of an artificial hand in the intact limb of healthy participants (Mulvey et al., 2012, 2015). TENS’ effect on enhancing another form of body representation, the rubber hand illusion, however, independent of the synchronous input of visuotactile sensation, might also reveal that TENS input proprioception secondary to the plastic change of body representation (Asao et al., 2019). Since rPMS and TENS have similar neurophysiological effects on human body but with minimal activation of cutaneous fibers (Beaulieu and Schneider, 2015), we propose in this study that it might be body representation that underlies response of proprioceptive acuity to rPMS.

This study had some limitations. First, to avoid the possible learning effect, repeated measurements at each extension position were only two times; therefore, we could not calculate proprioceptive precision for six extension positions, respectively. Second, we adopted one-session rPMS protocol, even though frequency of stimulation was up to 50 Hz and observed expected effects, but multiple sessions or longer stimulation time might achieve better performance. Third, our proposal of relating body schema and body image to proprioceptive bias and proprioceptive precision, respectively, might facilitate the understanding of this research field; however, it needs further experiments to provide more evidence, which is also our next study plan to prove their correlations.

## Conclusion

Different extension positions of the wrist joint could be divided into the lower-extension position and the higher-extension position according to proprioceptive bias. Moreover, replicated position in the lower-extension position tended to overshoot target position, while proprioceptive bias in the higher-extension position was more accurate. One-session rPMS could influence proprioceptive bias in the lower-extension position but not in the higher-extension position, and proprioceptive precision was not influenced at all extension positions.

By distinguishing the difference between proprioceptive bias and proprioceptive precision, we might attribute them to body schema and body image, respectively. To summarize, it might be body representation that underlies the response of proprioceptive acuity of wrist joint position sense to rPMS.

## Data availability statement

The raw data supporting the conclusions of this article will be made available by the authors, without undue reservation.

## Ethics statement

The studies involving human participants were reviewed and approved by Ethics Committee of Tohoku University. The participants provided their written informed consent to participate in this study.

## Author contributions

YX and SI: conceptualization. YX and KT: data acquisition and methodology. YX and MY: formal analysis and investigation. YX: funding acquisition, validation, and writing—original draft. SI: project administration, supervision, and writing—review and editing. All authors contributed to the article and approved the submitted version.
